# Screening for smoking and chronic obstructive pulmonary disease among employees of an electricity and gas company

**DOI:** 10.1192/j.eurpsy.2025.993

**Published:** 2025-08-26

**Authors:** I. Sellami, A. Abbes, R. Gargouri, R. Khemakhem, A. Feki, W. Feki, N. Bahloul, S. Kammoun, M. L. Masmoudi, M. Hajjaji, H. Ayadi, K. Jmal Hammami

**Affiliations:** 1 Occupationnal medecine; 2 Pneumology; 3 Rheumatology, CHU Hedi Chaker, Sfax, Tunisia

## Abstract

**Introduction:**

Chronic obstructive pulmonary disease (COPD) is a common and under-diagnosed disease. Screening for this disease and assessing smoking behaviour are essential to tobacco control measures.

**Objectives:**

Our study aims to screen for smoking and COPD among employees of an electricity and gas company.

**Methods:**

We conducted a descriptive, analytical, cross-sectional survey to screen for smoking and COPD among employees of an electricity and gas company. The survey was carried out during a COPD screening day, using a two-part questionnaire. Nicotine dependence was assessed using the Fagerström test. COPD screening was carried out using a COPD screening self-questionnaire from the French National Authority for Health.

**Results:**

Our population comprised 28 male participants. Active smoking was reported by 87.7% of participants. Nicotine dependence assessed by the Fagerström test was moderate to high in 54.7% of smokers. We found that 11 participants (39.2%), were identified as being “at risk” of developing COPD. Bivariate analysis showed that the COPD self-screening questionnaire score was associated with smoking and nicotine dependence.

**Conclusions:**

COPD and smoking among workers are prevalent in the electricity and gas company. Through this awareness-raising day, an action plan had prioritized anti-smoking programs directed at smoking employees to ensure their comprehensive care and prevent the onset of COPD.

**Disclosure of Interest:**

None Declared

